# Teprotumumab and sensorineural hearing loss: a propensity score-matched retrospective cohort study

**DOI:** 10.1530/EC-25-0293

**Published:** 2025-07-30

**Authors:** Maxim John Levy Barnett, Justin Lam, Mikaela Nikkola Jara-Tantoco, Carlo Casipit, Sarah Eidbo, Catherine Anastasopoulou

**Affiliations:** ^1^ Jefferson-Einstein Hospital, Internal Medicine, Philadelphia, Pennsylvania, USA; ^2^ Jefferson-Einstein Hospital, Endocrinology, Diabetes & Metabolism, Philadelphia, Pennsylvania, USA

**Keywords:** teprotumumab, sensorineural hearing loss, thyroid eye disease, Graves’ ophthalmopathy

## Abstract

Teprotumumab, a monoclonal antibody that antagonizes the insulin-like growth factor 1 receptor, is the first medication within the United States to receive Food and Drug Administration (FDA) approval for thyroid eye disease (TED). The FDA recently issued a warning regarding otologic complications, such as hearing loss. We aimed to study the association between teprotumumab and sensorineural hearing loss in thyrotoxic patients with TED. A real-world retrospective cohort study was performed through the TriNetX Collaborative Network (globally de-identified database) over a 5-year period. Inclusion criteria were patients at least 18 years of age with a diagnosis of Graves’ disease and TED, and were stratified into two cohorts: those treated with teprotumumab and those who were not. Propensity score-matching was performed for an even balance among cohorts (adjusting for age, sex, race, ethnicity, comorbidities, medications, smoking, and laboratory values), providing 532 patients per cohort. We identified a significantly higher risk difference of sensorineural hearing loss in patients treated with teprotumumab compared to those without exposure (risk difference 8.415%, 95% CI 4.954–11.876; 12.222 versus 3.808%), with a near three-fold increased relative risk for acquiring sensorineural hearing loss (risk ratio 3.21, 95% CI 1.936–5.323, *P* < 0.0001) over a 5-year period. Although the mechanism of teprotumumab-induced hearing loss remains unknown, and there is a lack of data to identify patients at risk (or those more likely to recover), our study confirms a higher risk for sensorineural hearing loss, which patients and providers alike must be aware of. Further studies providing prospective data are required to confirm such findings.

## Introduction

Thyroid eye disease afflicts up to 50% of patients with Graves’ disease and is known as Graves’ orbitopathy or ophthalmopathy (1). Numerous complications exist, with sight-threatening optic neuropathy being the most feared one. Thyroid eye disease is divided into an active and stable (fibrotic) stage, as evidenced by Francis Rundle’s curve, first noted in 1945 (2). Risk factors include smoking, evidence of active Graves’ disease, and a paradoxical worsening following radioiodine treatment. Treatment can involve selenium supplementation, steroids (oral or intravenous), rituximab, tocilizumab, and surgical decompression in the fibrotic stage (3). In January 2020, as a new therapy, the Food and Drug Administration (FDA) approved teprotumumab (trade name Tepezza) for the treatment of TED (4).

Teprotumumab, the first exclusively approved medication for TED, is a human IgG_1_ monoclonal antibody developed by Horizon Therapeutics (Ireland), initially for the management of oncological disease, but it never received approval for this (5). Subsequently, however, it was demonstrated to be of efficacy in thyroid eye disease. Teprotumumab antagonizes the receptor of insulin-like growth factor 1 (IGF-1), which is involved in the proliferation of orbital fibroblast and tissue expansion (5). Teprotumumab is administered intravenously every 3 weeks for a total of eight infusions, with an initial dose of 10 mg/kg followed by 20 mg/kg (for infusions two through eight) (6). Adverse effects are notable, including nausea, diarrhea, muscle spasms, hyperglycemia, and hearing impairment, among others; the latter adverse effect was added as a warning to the label by the FDA in July of 2023 (7).

With the paucity of the literature exploring this phenomenon and the increasing prescription prevalence of teprotumumab within the United States, we performed a retrospective cohort analysis reviewing the risk for developing sensorineural hearing loss in patients with TED treated with this medication.

## Materials and methods

### Study design

There are limited studies assessing the impact of teprotumumab-induced hearing loss; a significant proportion of the available data is derived from case reports and case series. Of the currently available studies, most are limited to certain geographic regions (Japan, the United States, or the European Union). Dissimilar to these previous studies, we used the TriNetX Global Collaborative Network (TriNetX, USA) to obtain de-identified healthcare records (electronically) of over 200 million patients in more than of 140 healthcare organizations in both the inpatient and outpatient settings; the purpose of this study was to provide global data, not limited by geographic region. At present, 21 countries are included in the TriNetX Network (African Continent: Ghana; Asia: Israel, Japan, Malaysia, Singapore, Taiwan, United Arab Emirates; Australian Continent: Australia; European Continent: Belgium, Bulgaria, Estonia, France, Germany, Italy, Lithuania, Poland, Spain, United Kingdom, Georgia; North American Continent: United States of America; South American Continent: Brazil). As de-identified patient data were used in a retrospective fashion, an institutional review board was not required for this study.

Using the 10th Revision of the International Statistical Classification of Diseases and Health Problems (ICD-10) codes (alongside the United States National Library of Medicine RxNorm codes), diagnoses and medications were appropriately identified on our platform. The population used in our study included both males and females (as well as those identified as ‘unknown gender’) who were at least 18 years of age, with the following ICD-10 codes: thyrotoxicosis with diffuse goiter (E05.0) and other disorders of the orbit (H05.89), or unspecified exophthalmos (H05.20), constant exophthalmos (H05.24), constant exophthalmos, bilateral (H05.243), displacement (lateral) of globe, left eye (H05.212), displacement (lateral) of globe, right eye (H05.211), displacement (lateral) of globe, bilateral (H05.213), extraocular muscle entrapment, unspecified (H50.68), extraocular muscle entrapment, unspecified, left eye (H50.682), extraocular muscle entrapment, unspecified, right eye (H50.681), or superior rectus muscle entrapment, unspecified eye (H50.679). Similarly, both inpatient and outpatient data were utilized. As mentioned, no geographic restriction was used for either cohort. Cohorts were separated based on those treated with teprotumumab (RxNorm 2274803) (cohort A) and those who did not have exposure to the medication (cohort B). The primary outcome of this study was to investigate the outcomes of sensorineural hearing loss (ICD-10: H90.3 and H90.5) in those with TED exposed to teprotumumab compared to those without exposure.

### Statistical analysis

The index event and time windows were defined to analyze patient outcomes. The index event was noted as the first date that patients fulfilled inclusion criteria for either cohort, with a time interval of 5 years to assess for sensorineural hearing loss. Data collection was obtained before and after matching. We employed one-to-one propensity score-matching to minimize confounding, using the greedy (nearest) neighbor matching algorithm with a 0.1 caliper of pooled standard deviation, allowing for an even balance among both cohorts. We matched for 18 variables (current age, age at index, gender, race, ethnicity, corticosteroids, thyroid modifiers, tobacco use, serum creatinine, serum thyrotropin, meningitis, diabetes mellitus, diuretics, aminoglycosides, and head injuries). The statistical method chosen for data analysis was the measure of association method, calculating a risk ratio and risk difference, with concurrent 95% confidence intervals and *P*-values (below 0.05 considered statistically significant). Those with the outcomes defined before the window was excluded from analysis. Furthermore, we incorporated an E-value measurement for the risk ratio to analyze if a potential unmeasured confounder could impact the strength of the association; an E-value of above 2.0 was considered moderately robust, and above 3.0 was considered strongly robust. All statistical analyses were conducted through the TriNetX platform.

## Results

**Figure 1:**
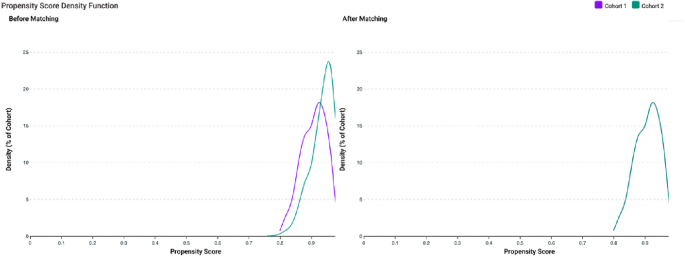
Before and after cohort characteristics with propensity-score matching.

The initial search obtained 77 healthcare organizations for cohorts A and B. Our initial search before matching identified *n* = 532 in cohort A and *n* = 8,628 in cohort B. Within cohort A, geographic distribution was 98% within the United States (and around 2% elsewhere, not further defined), and in cohort B, 94% within the United States (and 6% elsewhere, not further defined). Following one-to-one matching, we obtained *n* = 532 per cohort (total *n* = 1,064) (Fig. 1). After matching, cohort A demonstrated a mean age of 59.2 ± 14.7 compared to 60 ± 16 in cohort B. Further differences between cohorts A and B after matching included female gender (68.421 versus 70.301%), white race (69.925 versus 72.556%), tobacco usage (8.835 versus 8.083%), and serum thyroid-stimulating hormone (3.45 ± 10.1 mIU/mL versus 4.37 ± 17.5 mIU/mL) (Supplementary Table 1 (see section on Supplementary materials given at the end of the article)).

**Figure 2:**
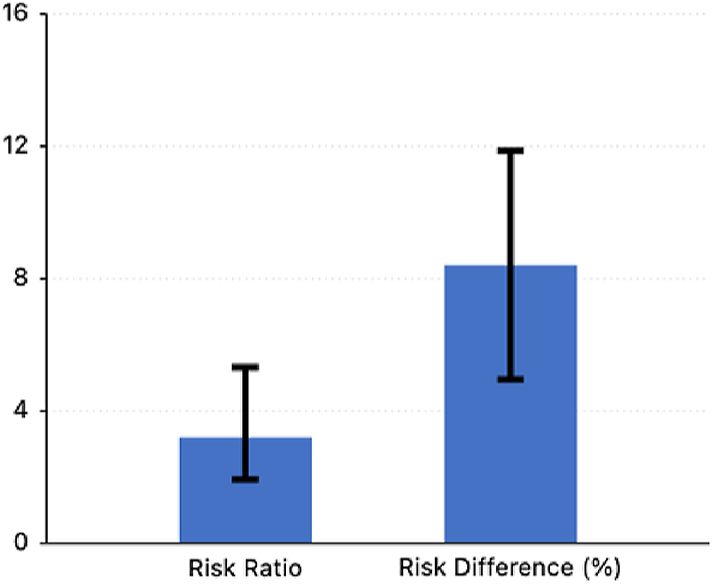
Risk ratio and relative risk of teprotumumab-related sensorineural hearing loss.

We identified a higher incidence of sensorineural hearing loss in cohort A (12.222%) compared to cohort B (3.808%), with a risk difference of 8.415% (95% CI 4.954–11.876) and a relative risk of 3.21 (95% CI 2.053–6.026, *P* < 0.0001), which was statistically significant (Fig. 2). We subsequently calculated an E-value of 5.87 from the risk ratio (strongly robust), suggesting unmeasured confounders would need to be associated with both teprotumumab and hearing loss by a risk ratio of at least 5.87 each to explain away the observed association, and a risk ratio of 3.52 to move the confidence interval to include the null (whereby no overall effect).

## Discussion

This retrospective study assesses the impact of sensorineural hearing loss in patients with TED. We found a significantly higher risk of hearing impairment with exposure. While the prevalence of hearing impairment in randomized controlled trials is estimated at around 10%, case reports and series have suggested the prevalence is higher than anticipated (6). Excluding case reports and case series, we identified *n* = 7 other studies that analyze the impact of hearing loss (with various definitions) in relation to teprotumumab (Supplementary Table 2) (8, 9, 10, 11, 12, 13, 14).

McGwin Jr and colleagues, alongside Zhao & Tao, both analyzed adverse events reported to the United States FDA Adverse Event Reporting System (FAERS) (15, 16). McGwin Jr and colleagues demonstrated a proportional reporting ratio (PRR) of 23.6 (95% CI % CI 18.1–30.8) for any hearing disorder, with subanalyses as follows: bilateral deafness (PRR 4.91 (95% CI 12.8–13.69)), eustachian tube disorders (PRR 34.9 (95% CI 4.9–247.4)), hypoacusis (PRR 10.1 (95% CI 7.6–13.3)), and tinnitus (PRR 8.7 (95% CI 6.2–12.1)) (15). Zhao & Tao noted adverse events relating to hearing loss with reported odds ratios as follows: tinnitus (ROR 39.92 (26.94–59.14)), deafness (ROR 106.77 (71.92–158.52)), ear discomfort (ROR 59.69 (30.82–115.59)), hypoacusis (ROR 17.73 (9.18–34.26)), ototoxicity (ROR 178.53 (56.46–564.54)), deafness unilateral (ROR 33.95 (8.44–136.59)), hyperacusis (ROR 31.06 (7.72–124.89)), deafness bilateral (ROR 52.86 (7.37–379.19)), ear disorder (ROR 17.41 (2.44–124.13)), autophony (ROR 2,907.62 (263.33–32,104.6)), and auditory disorder (ROR 43.72 (6.1–313.13) (16).

The definition of hearing impairment varied with each study, including sensorineural hearing loss (new or worsening), hypoacusis, hyperacusis, autophony, patulous eustachian tube, tinnitus, and ear fullness/pressure/plugging, with sensorineural hearing loss and hypoacusis being most common. Hearing impairment appears to occur after a mean of 3.8 infusions (between third to fifth infusion or 6–12 weeks); however, there is a wide range of reports, from third infusion to after completion (16 weeks) of therapy (17, 18). The literature is mixed about the perseverance of hearing loss upon discontinuation of teprotumumab, with a median remission of 50% (ranging from 33 to 100); tinnitus appears to be the most likely to resolve, followed by ear fullness/pressure/plugging (90%), with sensorineural hearing loss being the least likely to resolve (45.5%) (19). This is an important area of consideration, as around one-third of patients note a significant impairment in quality of life with reduction in their hearing, expressing regret about deciding upon treatment (20). Affected patients appear to be on average older (above 70 years), most prominent in the high and middle frequencies (21). A significant risk factor appeared to be pre-existing hearing loss (17). One report suggests that teprotumumab can increase the susceptibility of cochlear hair cells to noise-related trauma (as evidenced by sustained hearing loss following rifle blast) (22).

The underlying mechanism of action leading to hearing impairment (and other otological adverse events) is not fully understood; however, several theories exist. Most prominent is the notion of IGF-1 receptors being expressed in the cochlea. Preliminary trials have demonstrated a role of IGF-1 intratympanic injections for the treatment of refractory sensorineural hearing loss (although this has not been studied in patients whose hearing loss is related to teprotumumab) (23). Case reports have mixed evidence regarding corticosteroid administration; however, this is believed to be ineffective, as the underlying pathophysiology is unlikely to be inflammatory (23). Another consideration has been administering half-dose (10 mg/kg) for all eight infusions, which was noted in one case report to prevent the return of hearing loss in a patient who had previously experienced this during the first round of treatment (24).

Currently, there are no formal guidelines for audiologic screening in patients with thyroid eye disease undergoing treatment with teprotumumab. Highland and colleagues advise caution when using teprotumumab with other ototoxic agents or with noise exposure above 70 dB (25). Key and colleagues advise all patients to be counseled on the ototoxic properties of teprotumumab, and extra caution to be taken with those who have predisposing factors (19). Certain authors advise screening for all patients (especially at baseline to identify those at higher risk, such as pre-existent hearing loss), comparing to the standards of practice for other known ototoxic agents, such as cisplatin, with pre-treatment audiologic monitoring (19). Further recommendations include screening after treatment as well, to assess objective hearing function changes, while others advocate for screening before, during, and after treatment to identify patients who require prompt discontinuation by mid-treatment. Additional recommendations are to include patulous eustachian tube testing at baseline, as well as audiological testing (19). Those with baseline sensorineural hearing loss, on other ototoxic agents, exposure to occupational/recreational loud noises, or patients of older age should be advised of these heightened risks (19).

### Limitations

Notable limitations in this study are present, which must be considered when analyzing the results. This study assessed hearing impairment in patients with thyroid eye disease exposed to teprotumumab, which is a relatively small population, so generalizability to a larger population should be cautioned. Although our study assessed hearing impairment in patients with thyroid eye disease exposed to teprotumumab, it is important to note that thyroid eye disease itself is believed to be a risk factor for hearing impairment (with one study suggesting a prevalence of up to 11.1%) (26). Early data of teprotumumab analyzed for oncological diseases were not included in our study. Most of the trials assessing hearing impairment as an impact do not overtly express the ‘degree’ of hearing impairment (such as decibels and at which frequency hearing is most impaired). In these studies, there could be an underestimation due to missed cases that are not readily detectable without clinical investigation (but there could also be an overestimation if relying on subjective assessments). Furthermore, while some of the studies did list hearing loss separately from other otological adverse events, many failed to specifically separate hearing impairment from other otological features, due to hearing impairment being investigated as one overall outcome. We preferred to use TriNetX, which relies on correct clinician documentation and coding, which is often a source of error in electronic records. We were additionally unable to control the number of dosages of teprotumumab a patient received (or control for compliance), nor control the amount and duration of exposure to ototoxic medications, such as diuretics or aminoglycosides. Further research is required to determine if this finding remains statistically significant or if it is confounded by unmeasured variables. In addition, more research is required to investigate the underlying mechanism of hearing impairment, true likelihood of recovery, available treatment options, and a proposed screening guideline.

## Conclusion

Our retrospective analysis provides an estimated risk ratio for developing sensorineural hearing loss in patients with thyroid eye disease, which remained statistically significant with propensity score-matching. Further studies and data collation are required to answer the remaining questions, such as typical time to development of symptoms, number of dosages associated with adverse events, and even to investigate whether the sensorineural hearing loss remains permanent or can recover. Overall, our findings echo the warning by the Food and Drug Administration, and both clinicians and patients alike must be aware of this complication.

## Supplementary materials



## Declaration of interest

The authors declare that there is no conflict of interest that could be perceived as prejudicing the impartiality of the work reported.

## Funding

Publication was made possible, in part, by support from the Thomas Jefferson University Open Access Fund.

## Ethical approval

This paper is exempt from ethical committee approval as no human subjects were involved in the conduct of this research.
