# Supplementary material for: Teprotumumab and sensorineural hearing loss: a propensity score-matched retrospective cohort study
Source: Endocr Connect. 2025 Jul 30;14(7):e250293. doi: 10.1530/EC-25-0293 (PMC12323317; doi:10.1530/EC-25-0293)
Supplement: Supplementary file 1 [file supplementary_materials.pdf]

Table 1 - Before/After Propensity-Score Matching

Variables	Before Matching							After Matching						
	Cohort A	Cohort B	Patients (n) (Cohort A)	Patients (n) (Cohort B)	% of Cohort (Cohort A)	% of Cohort (Cohort B)	p-value	Cohort A	Cohort B	Patients (n) (Cohort A)	Patients (n) (Cohort B)	% of Cohort (Cohort A)	% of Cohort (Cohort B)	p-value
Current Age (mean ± Standard Deviations)	59.2 ± 14.7	56.3 ± 16.9	532	8,628	100%	100%	0.0002	50.2 ± 14.7	60 ± 16	532	532	100%	100%	0.3494
Age at Index	56.2 ± 14.6	53.4 ± 16.9	532	8,628	100%	100%	0.0003	56.2 ± 14.6	57 ± 16.1	532	532	100%	100%	0.3645
Not Hispanic or Latino	-	-	407	5,657	76.504%	65.566%	<0.0001	-	-	407	412	76.504%	76.444%	0.7158
White	-	-	372	4,668	69.925%	54.103%	<0.0001	-	-	372	386	69.925%	72.556%	0.3430
Female	-	-	364	6,446	68.421%	74.71%	0.0013	-	-	364	374	68.421%	70.301%	0.5060
Unknown Gender	-	-	25	282	4.699%	3.268%	0.0751	-	-	25	23	4.699%	4.323%	0.7677
Unknown Race	-	-	52	1,383	9.774%	16.029%	0.0001	-	-	52	54	9.774%	10.15%	0.8378
Male	-	-	143	1,900	26.88%	22.021%	0.0090	-	-	143	135	26.88%	25.376%	0.5767
Black or African American	-	-	61	1,702	11.466%	19.726%	<0.0001	-	-	61	58	11.466%	10.902%	0.7704
Glucocorticoids	-	-	398	4,897	74.812%	56.757%	<0.0001	-	-	398	396	74.812%	74.436%	0.8879
Thyroid Modifiers	-	-	419	5,491	78.759%	63.642%	<0.0001	-	-	419	431	78.759%	81.015%	0.3587
Diuretics	-	-	143	2,128	26.88%	24.664%	0.2507	-	-	143	145	26.88%	27.256%	0.8902
Aminoglycosides	-	-	118	1,389	22.18%	16.099%	0.0002	-	-	118	115	22.18%	21.617%	0.8240
Diabetes Mellitus	-	-	88	1,423	16.541%	16.493%	0.9766	-	-	88	-95	16.541%	17.857%	0.5696
Tobacco Use	-	-	47	490	8.835%	5.679%	0.0026	-	-	47	43	8.835%	8.083%	0.6594
Head Injury	-	-	23	420	4.323%	4.868%	0.5699	-	-	23	29	4.323%	5.451%	0.3936
Meningitis	-	-	10	24	1.88%	0.278%	<0.0001	-	-	10	10	1.88%	1.88%	1.0000
Creatinine (serum)	0.866 ± 0.498	0.958 ± 3.22	428	5,647	80.451%	65.45%	0.5567	0.866 ± 0.498	0.866 ± 0.482	428	420	80.451%	78.947%	0.9972
Thyrotropin (serum)	3.45 ± 10.1	4.71 ± 24.2	365	4,859	68.609%	56.317%	0.3242	3.45 ± 10.1	4.37 ± 17.5	365	358	68.609%	67.293%	0.3851

Table 2 – Characteristics of Included Studies

	Year Published	RR and 95% CI for Hearing Impairment	Criteria for Hearing Impairment	Hearing Outcomes of Affected Patients	Country of Origin	Total Number of Participants	% Female	Average Age (Years)	Study Participants	Follow-up Time	Study Design
Our Study	2025	RR = 3.21<br>95% CI 1.936-5.323<br>p < 0.0001<br><br>Teprotumumab: 55<br><br>Placebo: 19	Sensorineural hearing loss	Not Defined	Global	Total: 331<br>Teprotumumab: 450<br>Control: 949	Teprotumumab: 68.421%<br><br>Placebo: 70.301%	Teprotumumab: 56.2 ± 14.6<br><br>Placebo: 57 ± 16.1	Age ≥ 18 years with thyrotoxicosis with diffuse goiter and orbital disorders	5 Years	Retrospective Cohort Study over TriNetX Database
Hiromatsu et al. (8)	2025	RR = 4<br>95% CI 0.42-38.38<br>p = 0.169<br><br>Teprotumumab: 4/27<br><br>Placebo: 1/27	Patulous eustachian tube, neurosensory hypoacusis, tinnitus	Not Defined	Japan	Total: 54<br>Teprotumumab: 27<br>Placebo: 27	Teprotumumab: 67%<br><br>Placebo: Female: 74%	Teprotumumab: 46.6 ± 14.2<br><br>Placebo: 50.0 ± 13.4	Ages 20-80, Graves' disease, euthyroid or mild hypo/hyperthyroid state, CAS ≥ 3, moderate-to-severe TED, ≥ 3mm-increase in proptosis before TED onset and /or proptosis ≥ 18 mm at baseline, and TED duration ≤ 9 months.	24 Weeks	Multicenter Randomized, double-masked, placebo-controlled trial
Huang et al. (9)	2025	RR = 4.08<br>95% CI 1.32-12.62<br>p = 0.014<br><br>Teprotumumab: Not Defined<br><br>Placebo: Not Defined	Not Defined	Not Defined	USA + EU	Total: 232<br>Teprotumumab: 126<br>Placebo: 106	Not Defined	Not Defined	Not Defined	Not Defined	Systematic Review and Meta-Analysis
Markle et al. (10)	2024	RR = 2.85<br>95% CI 1.94-4.20<br>p < 0.001<br><br>Teprotumumab: 88/441<br><br>Placebo: 31/343	Tinnitus, sensorineural hearing loss, hypoacusis, hyperacusis, autophony, eustachian tube dysfunction	Not Defined	USA	Total: 884<br>Teprotumumab: 441<br>Placebo: 443	Not Defined	Not Defined	Diagnosis of thyroid eye disease and one or more ophthalmic manifestations within the last 20 years, and record of teprotumumab administration.	Not Defined	Retrospective Cohort Study over TriNetX Database
Douglas et al. (11)	2024	RR = 2.14<br>95% CI 0.51-8.93<br>p > 0.05<br><br>Teprotumumab: 6/41	Eustachian tube dysfunction, tympanic membrane disorder, tinnitus, hypoacusis, conductive deafness,	3/9 patients in the teprotumumab had recovered hearing/were recovering (not further defined)	USA	Total: 61<br>Teprotumumab: 41<br>Placebo: 20	Teprotumumab: 90%<br><br>Placebo: 76.2%	Teprotumumab: 49 ± 16.5<br><br>Placebo: 48.6 ± 14.4	Diagnosis of thyroid eye disease of 2-10 years duration, CAS ≤1 or no additional inflammation or progression in proptosis/diplopia for ≥ 1 year, proptosis ≥ 3mm from before	24 Weeks	Multicenter Randomized Controlled Trial

		Placebo: 2/20	unilateral deafness						TED and/or from normal, euthyroid/mildly hypo/hyperthyroid, no prior teprotumumab, and no steroids within 3 weeks of baseline.		
Kahaly et al. (12)	2021	RR = 25.1*<br>95% CI 1.45-434.5<br>p = 0.026<br>*Using Haldane-Anscombe Method<br><br>Teprotumumab: 12/84<br><br>Placebo: 0/46	Deafness, eustachian tube dysfunction, hyperacusis, hypoacusis, autophony	Not Defined	USA + EU	Total: 180<br>Teprotumumab: 84<br>Placebo: 86	Teprotumumab: 69%<br><br>Placebo: 77%	Teprotumumab: 51.5 ± 11.60<br><br>Placebo: 51.4 ± 13.09	Not Defined	Not Defined	Pooled data analysis, subgroup analysis, and off-treatment follow up of two multicentre, randomized, double-masked, placebo-controlled trials
Douglas et al. (13)	2020	RR = 11.2619*<br>95% CI 0.6426-197.3717<br>p > 0.05<br>*Using Haldane-Anscombe Method<br><br>Teprotumumab: 5/41<br><br>Placebo: 0/42	Hypoacusis, deafness, autophony, patulous eustachian tube	One patient had deafness (resolved) and two had hypoacusis (which resolved), one had autophony (Bilateral, resolved), and one had mild patulous eustachian tube (resolved)	USA + EU	Total: 83<br>Teprotumumab: 41<br>Placebo: 42	Teprotumumab: 71%<br><br>Placebo: 74% female	Teprotumumab: 51.6 ± 12.6<br><br>Placebo: 48.9 ± 13.0	Age 18-80 with a diagnosis of Graves' disease of active, moderate-to-severe thyroid disease, ocular symptoms began 9 months prior to baseline assessment and CAS of at least 4.	24 Weeks (With Extension to OPTIC-X trial; those not included in OPTIC-X were followed for 48 weeks).	Multicenter Randomized, double-masked, Phase 3 Placebo-controlled trial
Smith et al. (14)	2017	RR = 7.6*<br>95% CI 0.973-145.65)<br>p > 0.05<br>*Using Haldane-Anscombe Method<br><br>Teprotumumab: 3/43<br><br>Placebo: 0/44	Not Defined (Hearing Impairment and Tinnitus)	One with unilateral hearing impairment and one case of bilateral hearing impairment that resolved) alongside one case of tinnitus (unclear if resolved)	USA + EU	Total: 87<br>Teprotumumab: 43<br>Placebo: 44	Teprotumumab: 65%<br><br>Placebo: 82%	Teprotumumab: 51.6 ±10.6<br><br>Placebo: 54.2 ± 13.0	Age 18-75 with ophthalmopathy diagnosed no more than 9 months after onset of symptoms, CAS of 4 or more, no prior treatment apart from steroids.	48 Weeks	Multicenter Randomized, double-masked, placebo-controlled trial

Legend:

CI = Confidence Interval;

RR = Relative Risk
